# Microfluidic Irreversible Electroporation—A Versatile Tool to Extract Intracellular Contents of Bacteria and Yeast

**DOI:** 10.3390/metabo9100211

**Published:** 2019-09-30

**Authors:** Alexander Rockenbach, Suresh Sudarsan, Judith Berens, Michael Kosubek, Jaroslav Lazar, Philipp Demling, René Hanke, Philip Mennicken, Birgitta E. Ebert, Lars M. Blank, Uwe Schnakenberg

**Affiliations:** 1 Institute of Materials in Electrical Engineering 1, RWTH Aachen, 52074 Aachen, Germany; Alexander.Rockenbach@rwth-aachen.de (A.R.); judith.berens@rwth-aachen.de (J.B.); Kosubek.m92@googlemail.com (M.K.); jaroslav.lazar@rwth-aachen.de (J.L.); schnakenberg@iwe1.rwth-aachen.de (U.S.); 2 iAMB Institute of Applied Microbiology, ABBt-Aachen Biology and Biotechnology Department, RWTH Aachen, 52074 Aachen, Germany; sursud@biosustain.dtu.dk (S.S.); philipp.demling@rwth-aachen.de (P.D.); rene.hanke@rwth-aachen.de (R.H.); philip.mennicken@rwth-aachen.de (P.M.); birgitta.ebert@uq.edu.au (B.E.E.); 3 Current address: Novo Nordisk Foundation Center for Biosustainability, Technical University of Denmark, 2800 Kgs. Lyngby, Denmark; 4 Current address: Australian Institute for Bioengineering and Nanotechnology, The University of Queensland, 4072 Brisbane, QLD, Australia; 5 Bioeconomy Science Center BioSC, c/o Forschungszentrum Jülich, 52425 Jülich, Germany

**Keywords:** irreversible electroporation, microfluidics, microelectrodes, pulsed electric field electroporation, intracellular metabolites, enzymes, quenching, *E. coli*, *S. cerevisiae*

## Abstract

Exploring the dynamic behavior of cellular metabolism requires a standard laboratory method that guarantees rapid sampling and extraction of the cellular content. We propose a versatile sampling technique applicable to cells with different cell wall and cell membrane properties. The technique is based on irreversible electroporation with simultaneous quenching and extraction by using a microfluidic device. By application of electric pulses in the millisecond range, permanent lethal pores are formed in the cell membrane of *Escherichia coli* and *Saccharomyces cerevisiae*, facilitating the release of the cellular contents; here demonstrated by the measurement of glucose-6-phosphate and the activity of the enzyme glucose-6-phosphate dehydrogenase. The successful application of this device was demonstrated by pulsed electric field treatment in a flow-through configuration of the microfluidic chip in combination with sampling, inactivation, and extraction of the intracellular content in a few seconds. Minimum electric field strengths of 10 kV/cm for *E. coli* and 7.5 kV/cm for yeast *S. cerevisiae* were required for successful cell lysis. The results are discussed in the context of applications in industrial biotechnology, where metabolomics analyses are important.

## 1. Introduction

A major challenge in the field of systems biology is the quantitative description of the cellular metabolism, the synthesis and conversion rates (fluxes), and concentrations of metabolites. Quantification of intracellular metabolites allows capturing the status of the living cell, i.e., by providing insights into the regulation of the biochemical reactions of interest. This information helps in developing fundamental knowledge for, e.g., rational strain and bioprocess engineering [1,2,3,4]. One approach followed is the ‘stimulus response methodology’, where the response of the microorganism is characterized by measuring the intra- and extracellular concentrations of metabolites at high sampling rates after introducing a defined disturbance into the bioreactor [5,6,7]. In microorganisms, the necessity for high sampling frequency (as low as sub-second), fast inactivation of metabolic enzymes (quenching), and extraction of metabolites from the cells arises due to fast regulatory effects and high metabolite turnover, e.g., for ATP about 0.7–2.9 s. Metabolic inactivation is commonly achieved by a rapid change in temperature by mixing the biological sample with quenching solutions, e.g., perchloric acid at −20 °C, methanol at −70 °C, liquid nitrogen at −196 °C or hot water up to 100 °C [8]. The disadvantages of such methods are the dilution of the sample, contamination by solvents, and precipitation of salts during pH adjustment, or long heating times. A suitable alternative to overcome these challenges is a simultaneous inactivation and extraction of intracellular metabolites by short-time exposure of cells to high temperatures. By using, e.g., an integrated helical tube heat exchanger [9], the total content of the cells was released into the culture broth. Intracellular metabolite concentrations were then calculated by subtracting the metabolite content of filtered culture broth (the cell-free extracellular medium) from the total metabolite content after extraction [10]. Although the integrated simultaneous quenching and extraction procedure is suitable to measure intracellular metabolite concentrations, it shows limitations in (a) extracting thermolabile metabolites, (b) requiring high sample volumes, and (c) handling heat-tolerant microorganisms (e.g., hyperthermophilic bacteria). In summary, the field of metabolomics faces challenges in adopting a single procedure to quantify at least a usable fraction of intracellular metabolites [11].

Electroporation is a method for cell membrane permeabilization. When a cell is exposed to an external electric field of sufficient strength and duration, the cell membrane is permeabilized and pores are formed in the membrane. The damage caused can be reversible or irreversible depending on the parameters of the applied electric pulses and the recovery capability of the cells. Reversible electroporation is widely used in biotechnology and medicine for the transfer of drugs, DNA, and other biomolecules into the cells [12,13,14,15]. In contrast, irreversible electroporation (IRE) is induced when the applied electric field strength is high enough to increase the transmembrane potential beyond its critical value, approximately 0.5 to 1.0 V. The pores created cannot be sealed by the cell leading to dielectric breakdown and cell inactivation combined with the release of cell contents [16,17,18].

Beside high electric field strength, the irreversibility of the process depends on additional factors, such as the ionic composition of the medium, pH value, solution conductivity, temperature (Joule heating due to high electric field strength), pulse time, cell type, and cell size [19,20,21,22]. A literature survey showed that irreversible electroporation of *Escherichia coli* can typically be obtained by applying electric field strengths of 10–20 kV/cm within 10–40 pulses of 30–500 µs length [17,18,23,24,25,26,27,28]. In comparison, *Saccharomyces cerevisiae* needs most often less electric field strengths of 4–8 kV/cm with 4–10 pulses of 20–200 µs length to be electroporated irreversibly [26,29,30,31].

The concept of IRE can be applied in the field of metabolomics as an alternative method for the simultaneous extraction of metabolites from bacteria and cells [12,28,32,33]. To address small-scale reactors with cultivation volumes smaller than 100 mL, microfluidic approaches can favorably be applied for IRE applications because of their low reagent requirements. Microfluidic devices for electroporation are excellently reviewed in, e.g., [13,14,15,34,35,36,37,38,39,40,41]. Flow-through, trap-based, and electric field focusing constriction designs are typically used. Microfluidic approaches, in combination with microelectrodes embedded inside the microchannels, offer several advantages. Since the electric field is directly proportional to the voltage applied across the electrodes and inversely proportional to the distance between the electrodes, small distances between the microelectrodes allow a significant reduction of the applied potential, reaching electric field strengths in the range of kV/cm. Therefore, simple and cheap signal generators can be used to address the electrodes. Using microfluidic devices, reproducible cell handling as well as defined separation and detection of released components become easier as dilution is minimized.

Previous works on microfluidic devices for cell disruption indicate that the choice of proper electrode material is a significant challenge in irreversible electroporation. While high electric field strengths are necessary for cell disruption, electrode aging and electrolysis are heavily dependent on the pulse scheme. On the one hand, electrolysis occurs when the applied potentials exceed the redox potential of the liquid and on the other hand when the pulse duration is longer than the time taken to charge the electrical double layer. To overcome these challenges, electrode distances or channel confinements can be reduced as well as shorter pulses or bipolar pulses instead of unipolar pulses can be used [35]. Besides, increasing the resistance of the medium the cells are suspended in can also omit electrolysis [37]. Metal electrode materials like gold, chromium, silver-silver chloride, or platinum are commonly used for irreversible electroporation applications [35]. Open electrodes are favorable since a passivation layer acts as a high-pass filter, blocking low frequencies and the DC component [35]. Beside metal electrodes, polyelectrolytic gel electrodes [42] or three dimensional-shaped carbon electrodes [43] were also proposed. However, up to now, microfluidic-based IRE devices are applied only to dilute samples [17,24,25,37,44,45,46,47] or even single cells [48,49,50].

Several groups already published microfluidic devices with serpentine channel designs to enable electroporation, but only Filla et al. introduced a design for irreversible electroporation [51]. Two ink-printed carbon electrodes were placed near the inlet and outlet of the curved channel. Bovine aortic endothelial cells were seeded inside the channel using a highly time-consuming process before irreversible electroporation was carried out. Three other groups published continuous-flow devices with a curved channel for reversible electroporation applications. Rajabi et al. described a microfluidic chip, in which cells flew consecutively through five modules consisting of two split-and-recombine micromixers for biological system perturbation and chemical disruption, two stirring channels with embedded herringbone structures for chaotic advection used for reduced residence time distribution, and a spiral channel for efficient particle separation and media exchange [52]. Permeabilization at 37 °C and quenching at 4 °C were separated into two parts of the chip carried out with a noteworthy time delay. Wang et al. introduced a spiral-shaped microchannel in which Dean forces trigger vortex patterns [53]. In this arrangement, electroporation efficiency was significantly enhanced, because the entire cell membrane surface became uniformly permeabilized due to the exposure of a large fraction of the cell surface to the electroporation field. Finally, Wang et al. discussed a serpentine channel milled in an aluminum bar. Reversible electroporation was successfully achieved under continuous fluid flow using this sidewall electrode arrangement [54].

Here, we present as a proof-of-concept a microfluidic approach for IRE applied directly to cell suspensions samples with the novel combination of simultaneous inactivation and extraction for monitoring intracellular metabolites. High-throughput sampling is enabled with a sampling rate of 2.5 mL/h, which contrasts conventional rates of around 0.06 mL/h in typically used flow-through microfluidic devices. By application of microsecond pulses and electric field strengths of about 10 kV/cm, successful disruption of bacteria and yeast cells were observed. The release of the intracellular compound glucose-6-phosphate (G6P) in the electroporated samples demonstrate the application of the developed microfluidic device for both monitoring the concentration of intracellular metabolites and the metabolic activity in microorganisms.

## 2. Results

### 2.1. Chip Fabrication

**Figure 1:**
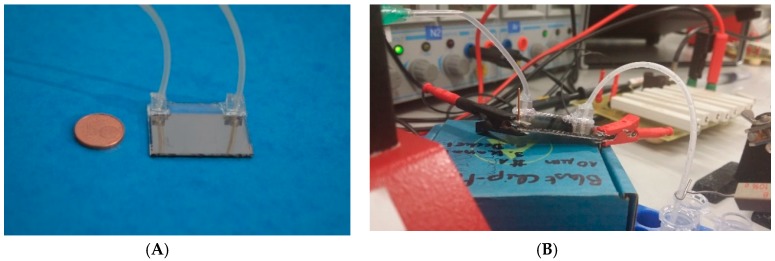
(**A**) Manufactured chip for irreversible electroporation. Two glass slides with platinum metallization are bonded together. A meander-type microfluidic channel is embedded between the two slides. Inlet and outlet tubes are connected via custom-made poly(dimethylsiloxane) (PDMS) connectors. (**B**) Experimental setup. The inlet tube is on the left side of the chip; the outlet tube on the right feeds directly into an Eppendorf tube (here not ice-cooled). In the background, the board (right) and the electronic equipment is shown. For both pictures: Footprint of the chip: 32 × 26 mm.

Figure 1 shows the manufactured chip with tubes connected via custom-made poly(dimethylsiloxane) (PDMS) connectors to contact inlet and outlet as well as the experimental setup.

### 2.2. Irreversible Electroporation

Well-grown cell suspensions of *E. coli* and *S. cerevisiae* were diluted to an optical density (OD_600_) of 1. The syringe pump continuously pumped cell suspensions into the microfluidic chip. Electroporated cell samples were collected for various assays from the outlet tube. The cell disruption efficiency was estimated from the plating and fluorescence assays as described above.

**Figure 2:**
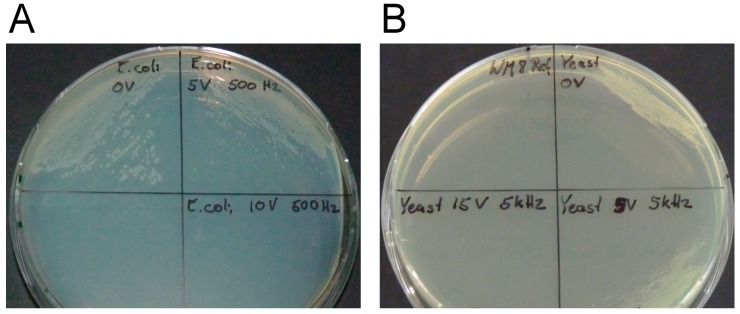
The influence of different applied electric field strengths on the viability of *Escherichia coli* (**A**) and *Saccharomyces cerevisiae* (**B**) shown from plating assay. For *E. coli*, the viability assay shows results after one day of plate incubation at 37 °C of electroporated samples from the chip. Cells were found to have grown in the control experiment (0 kV/cm, top left (**A**)) and the 5 kV/cm sample (top right (**A**)) and no growth was observed in the 10 kV/cm sample (bottom right (**A**)) indicating successful irreversible electroporation. For *S. cerevisiae*, the viability assay shows results after one day of plate incubation at 30 °C of electroporated samples from the chip. Cells were found to have grown in the control experiment (0 kV/cm, top right (**B**)) and the 2.5 kV/cm sample (bottom right (**B**)) and no growth was observed in the 7.5 kV/cm sample (bottom left (**B**)) indicating successful irreversible electroporation.

The results of the plating and fluorescence assay for *E. coli* and *S. cerevisiae* are presented in Figure 2. With respect to the *E. coli* viability assay, many colonies grew after an applied voltage of 0 kV/m, whereas a reduced vitality was observed for an applied electroporation voltage of 5 kV/m. For 10 kV/cm, no *E. coli* colonies were observed. These results correlate well to the fluorescent microscopy assay (Supplementary Materials; Figure S1). Living bacteria with an intact cell membrane are stained in green, whereas the cells in the 10 kV/cm sample are stained in red, corresponding to cells with damaged cell membranes. The *S. cerevisiae* cells were disrupted at an applied electric field strength of 7.5 kV/cm (Figure S2). In the fluorescent microscopy assay, differences were observed in the number of observed *E. coli* (Figure S1) and *S. cerevisiae* (Figure S2) cells. These differences can be attributed to both insufficient sensor sensitivity and sample magnification.

### 2.3. Enhanced Release of Intracellular Contents with Increasing Field Strength

**Figure 3:**
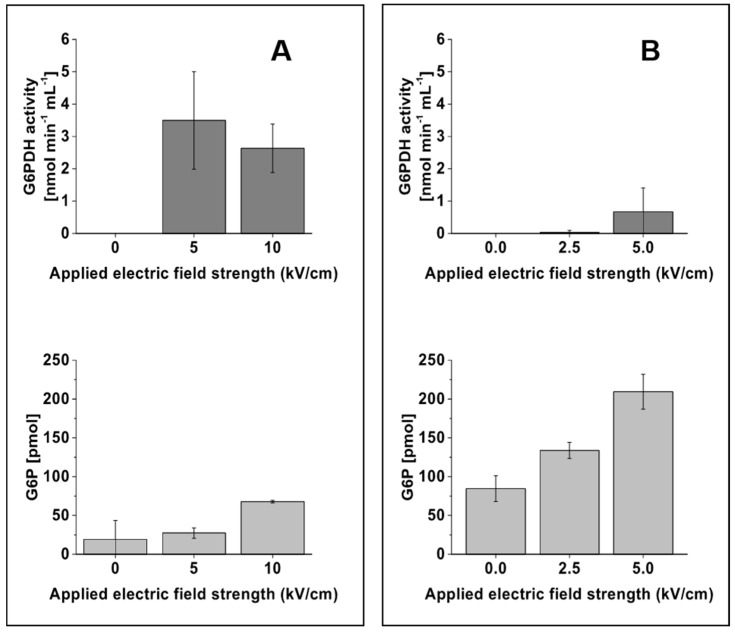
Influence of different electric field strengths on the activity of the enzyme glucose-6-phosphate dehydrogenase (G6PDH) and the amount of glucose-6-phosphate (G6P) from *E. coli* ((**A**), left column) and *S. cerevisiae* ((**B**), right column). The dark and light grey colored bar charts denote, respectively, the activity of the enzyme G6PDH and the amount of G6P at the given electric field strengths. The data were obtained from three biological replicate experiments performed with three different microfluidic chips. The error bar represents the standard deviation calculated from the biological replicates.

The electroporated samples leaving the microfluidic device were processed and analyzed for the release of intracellular contents. The substrate glucose is taken up by the cell and converted into glucose-6-phosphate (G6P), which is then oxidized by the enzyme glucose-6-phosphate dehydrogenase (G6PDH), the first enzyme in the pentose phosphate pathway, to 6-phosphogluconate. Hence, to observe and validate the release of the intracellular content, the activity of the enzyme G6PDH and the concentration of the intracellular compound G6P were analyzed in the electroporated samples. The release of the intracellular metabolite G6P was found to correlate with the applied electric field strength for both *E. coli* and *S. cerevisiae*. With increasing field strengths, the electroporated cells secreted higher amounts of G6P, and a maximum amount of G6P was observed with an applied field strength of 10 kV/cm for *E. coli* and 5 kV/cm for *S. cerevisiae* (Figure 3A,B). However, the activity of the enzyme G6PDH was found to have no significant correlation with increasing electric field strength. Due to the higher standard deviation observed in G6PDH activity with *S. cerevisiae* cells (Figure 3B, top), an increase of enzyme activity with increasing electric field strengths cannot be validated from this study. The data rather indicate that the applied electric field strengths are sufficient to quench the activity of enzymes in *S. cerevisiae*. In contrast, the enzyme G6PDH in the *E. coli* samples was found to be active at 5 and 10 kV/cm electric field strengths (Figure 3A, top), indicating the need to increase the field strength to guarantee quenching in this organism.

## 3. Discussion

Our study provides a proof-of-concept for the use of the developed chip-based setup for the extraction of intracellular contents via irreversible cell electroporation. While here tested with dilute cell suspensions (OD_600_ ~ 1) supplied with a syringe pump, the device can easily be reconfigured for direct sampling from a bioreactor and cell suspension of higher density. The approach simplifies experimental handling and can enable on-line monitoring of metabolites over time with high sampling rates. The setup is extremely favorable in comparison to other approaches, in which high efforts must be made to ensure proper dilution of the medium before electroporation [27,55,56]. The results demonstrate that electric field strengths of 10 kV/cm for *E. coli* and 7.5 kV/cm for yeast *S. cerevisiae* are sufficient to extract the intracellular contents from the cells. These values lie within the above-mentioned parameter ranges for both cell types indicating that a lower electric field strength and pulse length is needed for irreversible electroporation of *S. cerevisiae.* The type of microorganism affects the inactivation of microorganisms. Generally, yeasts are more sensitive to pulsed electric fields than Gram negative bacteria which are more sensitive than Gram positive bacteria, as Geveke et al. pointed out [57]. The lower field strength required to irreversibly electroporate *S. cerevisiae* vs. *E. coli* can be explained by several factors: first, the extra transmembrane potential of 0.5–1.0 V, which is needed for irreversible electroporation, is linearly proportional to the electric field strength and the cell diameter [35]. Therefore, the required field strength is an inverse function of the cell diameter. *S. cerevisiae* has a roughly 4-fold larger diameter than *E. coli*, significantly reducing the required field strength. Second, the medium used to cultivate *S. cerevisiae* contained besides mineral salts 10 g/L sodium glutamate. The ionic strength of this medium is much higher than the mineral salt medium M9 used for *E. coli*. The higher ionic strength increases the conductivity and thereby reduces the voltage/field strength that needs to be applied for IRE. Third, the lower pH of the *S. cerevisiae* medium (5 vs. 7 of *E. coli*) further contributes to the conductivity and reduces the required voltage.

The experimentally obtained data can be compared to data performed in standard commercially available electroporation cuvettes because of similar planar layouts, especially in electrode distances and area, respectively. The prototype of the electroporation cuvette was proposed by Potter et al. in 1984 [58] and investigated in detail with regard to electrical field strength and cuvette heating by Pliquet et al. in 1996 [59]. In cuvettes, *S. cerevisiae* can be successfully inactivated at electric field strength between 7.5 and 16 kV/cm and 1–1000 pulses of 0.01–0.3 ms duration [57,60,61], whereas for *E. coli*, irreversible electroporation was successfully obtained at electric field strength between 5 and 50 kV/cm with 20–100 pulses of 0.002–0.3 ms length [28,62,63,64,65]. This compilation shows that our results obtained from the microfluidic chip also fit the parameter ranges for cuvettes. As discussed above, is also confirmed here that for cuvettes higher electric field strength must be applied for *E. coli* than for *S. cerevisiae* for successful irreversible electroporation. The major advantage of using the microfluidic setup instead of cuvettes is the possibility to address continuous flow applications with online irreversible electroporation combined with quenching.

The hypothesis for quenching is due to the heat generated during electroporation. During the electroporation experiments, the generation of bubbles inside the channels assumes that temperatures near the boiling point were induced.

Microfluidic devices to electroporate and extract intracellular contents can be found useful in the field of microbial biotechnology and quantitative metabolomics [4,5]. Bioreactor-coupled integrated sampling procedures [9] are highly valuable in reducing the number of unit operations involved in sample processing and thus to avoid the bias in the quantification of intracellular metabolite concentrations [11]. The developed microfluidic device in this study allows integration into mini-bioreactor systems [66,67,68], thus enabling parallelized cultivations to elucidate microbial physiology. The device layout can be scaled to handle high-volume cell samples. Coupling of such a microfluidic device to a high-resolution mass spectrometer might be useful to understand the metabolic fluxes in engineered microorganisms by performing isotope labeling experiments [69,70] and for robust real-time metabolome profiling, which allows monitoring of the dynamics of metabolic processes in different microorganisms [71].

## 4. Materials and Methods

### 4.1. Design of a Microfluidic Device for Irreversible Cell Electroporation

**Figure 4:**
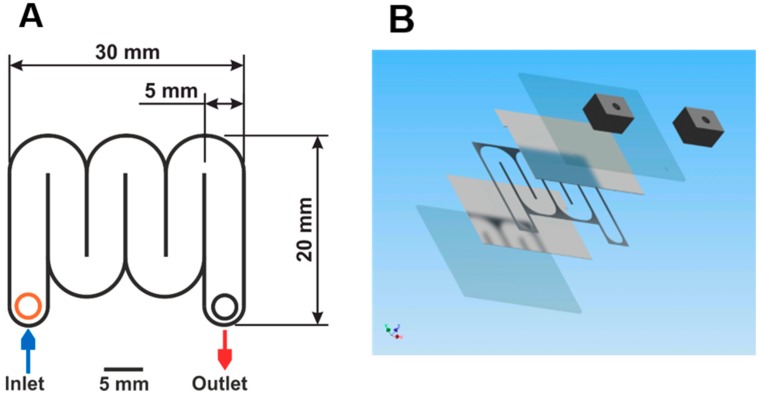
(**A**) Top view onto the microfluidic chip. The meander-type channel has a width of 5 mm, a length of 120 mm, and a height of 10 or 20 µm. Top and bottom of the channel consist of glass slides covered with platinum. Overall chip dimensions: 30 × 20 × 1 mm. (**B**) Exploded 3D view of the chip. From bottom to top: glass substrate, platinum layer, SU-8 with a serpentine channel, platinum layer, glass substrate, PDMS connectors for inlet and outlet.

To enable IRE and simultaneous quenching of cells, a microfluidic chip was developed. The schematic layout is shown in Figure 4. The chips were fabricated using microsystem technologies. Glass wafers with a thickness of 300 µm thick were coated on one side with an adhesion layer of 30 nm titanium followed by a layer of 400 nm thick platinum using a magnetron sputtering machine (Nordiko 2550, Havant, UK). A sheet resistance of 0.77 Ω/sq was obtained. Platinum was chosen as electrode material because of its well-known resistance to corrosion. On one of the wafers, a SU-8 layer (MicroChem Corp., Westborough, MA, USA), was deposited and structured by standard UV-lithography (MA6, Suss Microtech, Munich, Germany) defining the microfluidic channel. The channel length needed to obtain cell disruption can be calculated. As mentioned above, significant irreversible electroporation of both cell types under investigation was typically reached after around 10 pulses when a sufficiently high electric field strength was applied. To ensure sufficient warm-up of the chip for simultaneous inactivation of biomolecules, we increased the number of pulses to 100. The length L of the microfluidic chamber, in which these 100 pulses can be applied, depends on the volumetric flow rate and can be calculated by the equation L = (n_pulse_ × V_flowrate_)/(H × W × f), with n_pulse_ the number of pulses, L, H, and W length, height, and width of the microfluidic channel, respectively. V_flowrate_ is defined as the volumetric flow rate and f as the frequency of electric field pulses, respectively. Based on the equation, the channel length was calculated to be 120 mm. The channel width was specified to 5 mm. Blocking of the channel by the cultivation broth was prevented by defining two different channel heights: chips with channel heights of 10 µm were used for the broth containing the small-sized *E. coli* cells, while a gap of 20 µm between the two glass slides was sufficient to process the broth with larger *S. cerevisiae*. With these channel heights, channel volumes of 0.6 µL for experiments with *E. coli* and 1.2 µL for the experiments with *S. cerevisiae* were obtained, respectively. Holes for inlet and outlet were drilled in the opposite wafer. After dicing into 32 × 26 mm chips, a wafer bonder SB6e in combination with a mask aligner MA6 (both Suss Microtech) was used to align and bond two corresponding chips. After gluing, 5 × 5 × 8 mm custom-made poly(dimethylsiloxane) (PDMS) connectors to the inlet and outlet holes as well as Teflon tubes with an inner diameter of 100 µm were connected.

**Figure 5:**
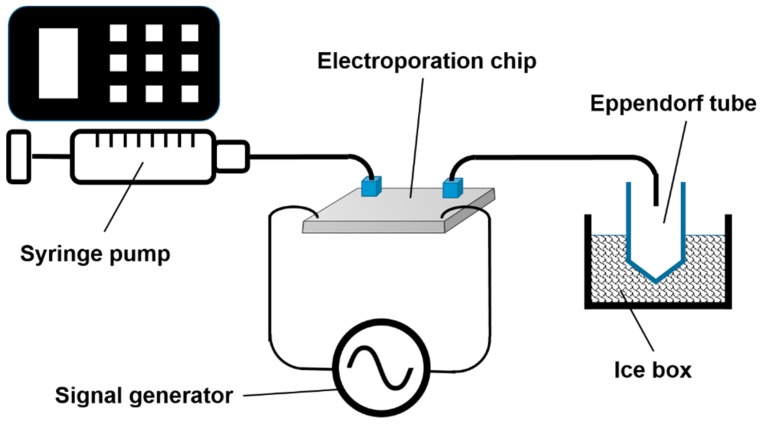
Schematic drawing of the measurement setup. For the sake of clarity, the analog circuit board, as well as the two oscilloscopes, are not shown. Drawing is not to scale.

The inlet tube was connected to the syringe pump with cell suspension, whereas the outlet tube feeds the broth directly into ice-cooled Eppendorf tubes, as depicted in Figure 5. Prior to the start of an experiment, the electrodes were connected to two oscilloscopes (TDS 2024B and TD S2024B, both Tektronix, Beaverton, OR, USA) and a function generator PM5134 (Philips, Hamburg, Germany) via a custom-made analog circuit board, which guaranteed a fast rise of the voltage at the chip, even when conductive fluid was used.

### 4.2. Application of Electric Pulses

Three AC voltages of 5, 10, and 15 V, respectively, were applied to the electrodes. For the chip used for electroporation of *E. coli*, the voltages corresponded to electric field strengths of 5, 10, and 15 kV/cm whereas for the chip used for *S. cerevisiae* the applied voltages were related to field strengths of 2.5, 5, and 7.5 kV/cm, respectively. Best irreversible electroporation was obtained with an applied frequency of 500 Hz and a pulse length of 200 µs for *E. coli* and 5000 Hz and a pulse length of 20 µs for *S. cerevisiae*, respectively. During the pulses, the maximum of the electric field strengths was obtained between the electrodes. The identified best parameter ensures rapid warm-up of the chip necessary for simultaneous inactivation of intercellular molecules. Due to the observed formation of vapor in the chip, the temperature in the channel reached boiling temperature.

### 4.3. Microbial Organisms, Media, and Cultivation Conditions

The *Escherichia coli* K-12 strain BW25113 [72] and the *Saccharomyces cerevisiae* strain CEN.PK 113-1A (EUROSCARF, Frankfurt, Germany) were used throughout this study. For experiments with *E. coli*, cells stored in cryo stocks (15% w/v glycerol) at −80 °C were recovered on Lysogeny Broth agar plates and grown for 24 h at 37 °C. A single colony was transferred into 10 mL liquid Lysogeny Broth medium [73] in culture tubes for the first pre-culture. The culture was incubated at 37 °C and 200 rpm for 6–8 h (Ecotron, Infors HT, Germany). Approximately, 500 μL of this culture was transferred to the subsequent second pre-culture consisting of 50 mL standard M9 mineral salt medium [73] with 5 g/L glucose as a carbon source and cultivated at 37 °C and 200 rpm. A well-grown overnight culture was washed twice with M9 medium and diluted with M9 medium to an approximate optical density (OD_600_) of 1.

Cell solutions of *S. cerevisiae* were prepared accordingly but were recovered on YPD agar (YPD + 2% Agar) plates, and the single colony was transferred into 10 mL liquid YPD medium [73]. To enable optimum growth of *S. cerevisiae*, the cultivation temperature was lowered to 30 °C. Here, 50 mL WM8 mineral salt medium [74] with 5 g/L glucose was used.

### 4.4. Sample Processing

For the irreversible electroporation experiments, 5 mL of the respective culture was loaded into the syringe pump and transferred into the chip with a flow rate of 2.5 mL/h. The samples leaked from the outlet were cooled in an Eppendorf tube in an ice bath maintained at a temperature of 0 °C. Approximately 1 mL sample was collected from the tube and processed for the following assays: (a) plating assay, (b) fluorescence microscopy assay, (c) glucose-6-phosphate assay, and (d) glucose-6-phosphate dehydrogenase enzyme assay.

### 4.5. Viability Tests

To access the cell viability after the electroporation event, 5–10 µL of the electroporated sample were immediately streaked onto Lysogeny Broth agar plates (for *E. coli*) and YPD agar plates (for *S. cerevisiae*) and incubated at 37 °C (for bacteria) and 30 °C (for yeast) for 24 h.

### 4.6. Fluorescence Microscopy Test

The extent of cell permeabilization was determined qualitatively by using a fluorescent dye assay (L-7012 LIVE/DEAD BacLight Bacterial Viability Kit, MoBiTec, Göttingen, Germany) in combination with fluorescence microscopy. Briefly summarized, 20 µL of the sample was mixed with 20 µL dye mix containing 10 µM of STYO9 and 60 µM propidium iodide and incubated for 15 min in the dark. Note: the collected samples from the chip outlet were immediately processed to prevent cell death and thus a biased result from the live/dead assay. A volume of 5 µL per sample was transferred on a microscope slide. Fluorescence images were taken using a Leica AF 6000 LX video microscope including LAS AF 3.2.0.9652 software (Leica Microsystems CMS GmbH, Germany) and using an HCX PL APO 40x/1.10 WATER objective in combination with an HC PL APD 63x/1.40-0.60 OIL objective.

### 4.7. Glucose-6-Phosphate (G6P) and Glucose-6-Phosphate Dehydrogenase (G6PDH) Assay

To quantify the amount of G6P released and determine the activity of the enzyme G6PDH, 1 mL of the electroporated samples were treated using the following kits: (a) High Sensitivity Glucose-6-phosphate Assay kit (MAK021, Sigma Aldrich, St. Louis, MO, USA) for G6P quantification and (b) Glucose-6-Phosphate Dehydrogenase Assay Kit (MAK015, Sigma Aldrich, St. Louis, MO, USA) for G6PDH activity determination.

## 5. Conclusions

As a proof-of-concept, a microfluidic serpentine channel made of SU-8 on a glass chip was developed for metabolomics sample preparation by irreversible electroporation. In a constant stream of cell suspension, both *E. coli* and *S. cerevisiae* were disrupted entirely, and the concept of sampling and inactivation was shown here with a G6P and G6PDH assay, by live-dead staining, and plating on agar plates. All methods showed successful disruption at electric fields of 10 kV/cm for *E. coli* and 7.5 kV/cm for *S. cerevisiae*. The proposed novel method has the advantage of rapid metabolite harvest using a single device. The ability to operate the device continuously allows the use of small-scale continuous fermenters, previously unsuited for metabolomics analyses. Irreversible electroporation, as presented here, might be used to simplify sample preparation for metabolomics and thereby improve the quality of this high content technique.

## Supplementary Materials

The following are available online at https://www.mdpi.com/2218-1989/9/10/211/s1, Figure S1: The influence of different applied electric field strengths on the viability of *E. coli*, Figure S2: The influence of different applied electric field strengths on the viability of *S. cerevisiae*
