# Supplementary figures and images for: Microfluidic Irreversible Electroporation—A Versatile Tool to Extract Intracellular Contents of Bacteria and Yeast
Source: Metabolites. 2019 Sep 30;9(10):211. doi: 10.3390/metabo9100211 (PMC6835232; doi:10.3390/metabo9100211)

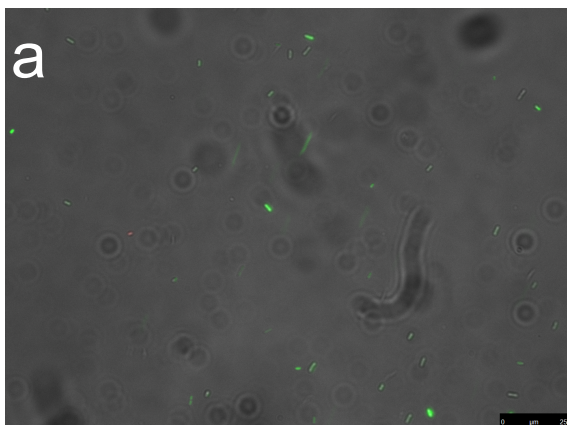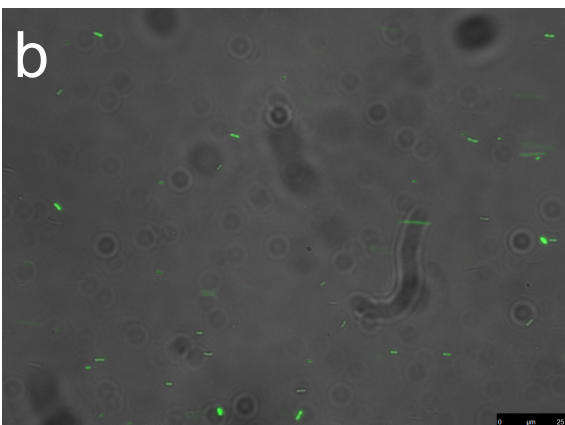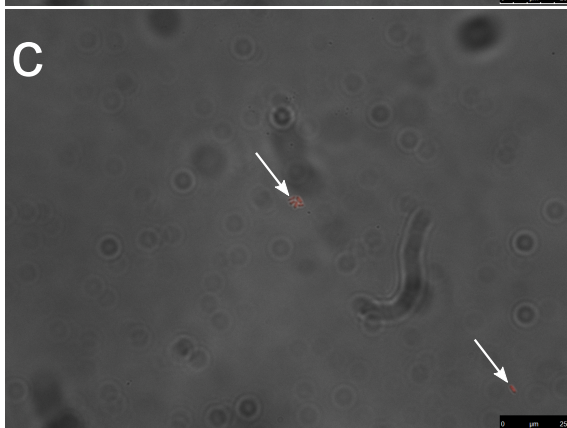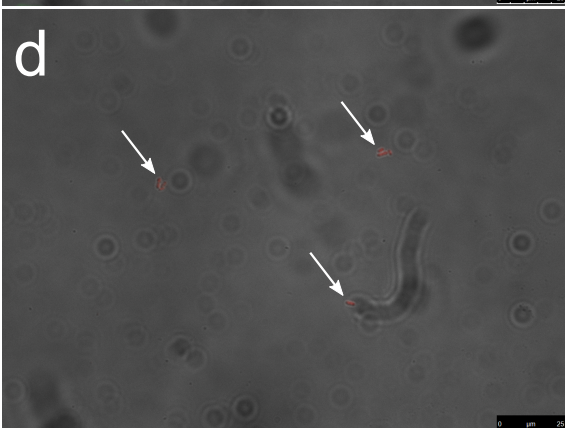

Supplement: Supplementary file 1 [file metabolites-09-00211-s001.zip › supplementary materials/Figure S1_Ecoli microscopy.pdf]

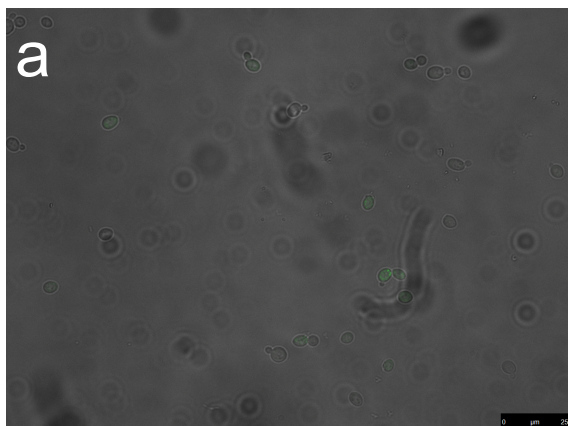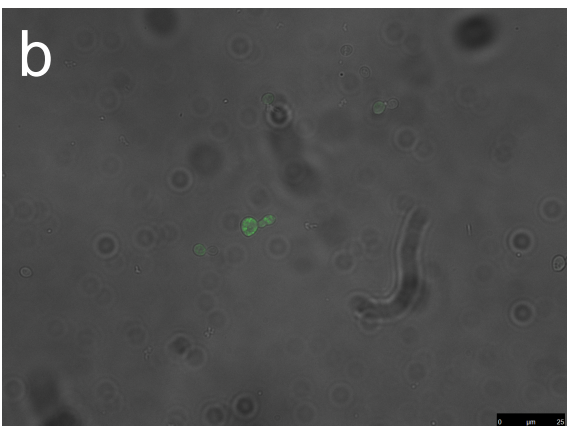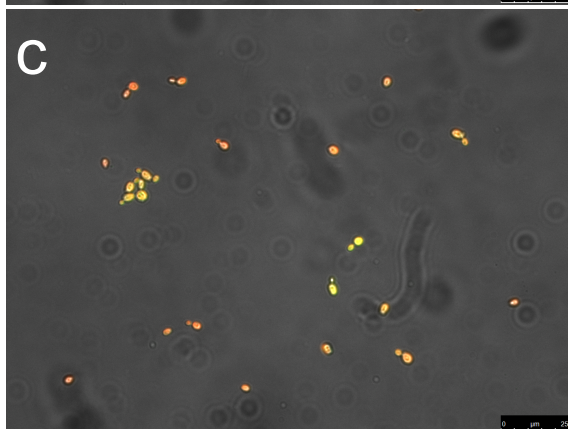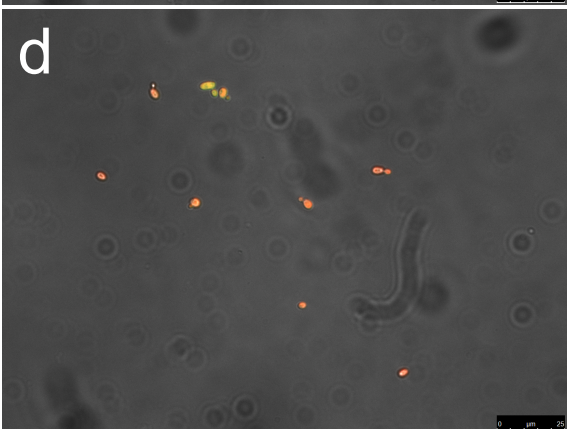

Supplement: Supplementary file 1 [file metabolites-09-00211-s001.zip › supplementary materials/Figure S2_Yeast microscopy.pdf]
